# Krukenberg tumor as an incidental finding in a full-term pregnancy: a case report

**DOI:** 10.1186/s13256-021-02875-6

**Published:** 2021-05-29

**Authors:** Felipe Mendoza-Rosado, Orlando Nunez-Isaac, Alan Espinosa-Marrón, Katheryn Lopez-Arjona, Fernando Davila-Martinez

**Affiliations:** 1 Research Department, Hospital General Dr. Manuel Gea González, Mexico City, Mexico; 2 Health Sciences School, Universidad Marista de Merida, Merida, Mexico; 3 Department of Nutrition, Harvard T.H. Chan School of Public Health, Boston, MA USA; 4 Department of Gynecology and Obstetrics, Hospital Regional Ignacio García Téllez, Merida, Mexico

**Keywords:** Krukenberg tumor, Neoplasm metastasis, Ovarian neoplasms, Female urogenital diseases and pregnancy complications, Case report

## Abstract

**Background:**

Krukenberg tumor is a rare metastatic tumor of the ovary with histopathological features known as signet ring cells. It usually develops in women around 45 years of age. However, here we describe an uncommon case in a 38-year-old pregnant woman. We report this case due to our unusual findings, the uncommon presentation in this younger age bracket, its diagnostic challenge, and poor prognosis.

**Case presentation:**

We describe a unique case of a young Mexican woman with a history of painful vaginal bleeding at 13 weeks of pregnancy and treated for abruptio placentae. In her routine prenatal visit at week 20 of pregnancy, she was found to have a uterine fundus exceeding the expected measure for her gestational age and was referred to the hospital to discard polyhydramnios. Upon admission, a pelvic ultrasound was performed displaying normal findings in a 25-week pregnancy, and also showing bilateral enlarged ovaries with heterogeneous echogenicity. Magnetic resonance imaging revealed a left tumoral lesion with dimensions of 22.1 × 13.6 × 16.3 cm presenting regular lobulated contours with displacement of peripheral structures and mild compression of the bladder, the left ureter, and the inferior vena cava. The lesion was heterogeneous with irregular borders. The patient was scheduled for a cesarean section; during the operation, the abdominal cavity showed bilateral tumors compatible with MRI findings. The ovarian tumors were sent to pathology, and the results showed poorly differentiated mucinous adenocarcinoma (World Health Organization grade III) with extensive signet ring cells, indicative of a Krukenberg tumor.

**Conclusion:**

This case report describes an uncommon example of a young pregnant woman without identifiable risk factors for gastric cancer who manifested a Krukenberg tumor. This incidental finding suggests that pregnancy obscured the cancer’s clinical appearance. The rapid deterioration in the patient’s condition corresponds to what is described in the literature. The limited information regarding this neoplasm in Mexico and the torpid evolution of the case highlight the importance of conducting additional studies to generate therapeutic interventions aimed at increasing overall survival.

## Background

Krukenberg tumor (KT) is a rare tumor of the ovary, characterized by poor prognosis. The tumor was named after Friedrich Ernst Krukenberg, who first described five cases of a new ovarian malignancy in 1896. Years later, it was discovered that KTs are ovarian metastases secondary to specific malignancies (signet ring cell carcinomas), most of which derive from the gastrointestinal tract. The stomach was previously reported to be the most common primary site, followed by the colon, appendix, and breast. Recent literature reveals an increased incidence of tumors originating from the colon. Of all ovarian tumors diagnosed, KTs make up about 1–2% [1].

The tumors are often bilateral (over 80%), given their metastatic nature. The average age of diagnosis is 45 years. Symptoms of KT can be very nonspecific, one of the most common being abdominal distension, which in pregnancy can be due to non-pathological components of pregnancy such as fat, fluid, fetus, flatus, or feces. A physician should also consider other causes such as sepsis, organ failure, and tumors. Specifically, symptoms of KT typically manifest due to ovarian involvement and mass effect, which causes abdominal pain, bloating, nonspecific gastrointestinal symptoms, or ascites with malignant cells [2, 3]. The diagnosis of KT is currently based on the World Health Organization (WHO) diagnostic criteria considering Serov and Scully’s pathological description as ovarian tumors with the presence of mucus-filled signet ring cells (SRCs) accompanied by a sarcoma-like proliferation of the stroma [4].

## Case presentation

### Chief complaints

A 38-year-old Mexican female patient attended the hospital with a pregnancy of 20 gestational weeks (according to her last menstrual period) for a routine prenatal visit. The patient was only complaining of abdominal fullness. During the physical examination, she was found to have a uterine fundus exceeding the expected measure for her gestational age. She was therefore referred to our hospital for further assessment.

### History of present illness

**Fig. 1:**
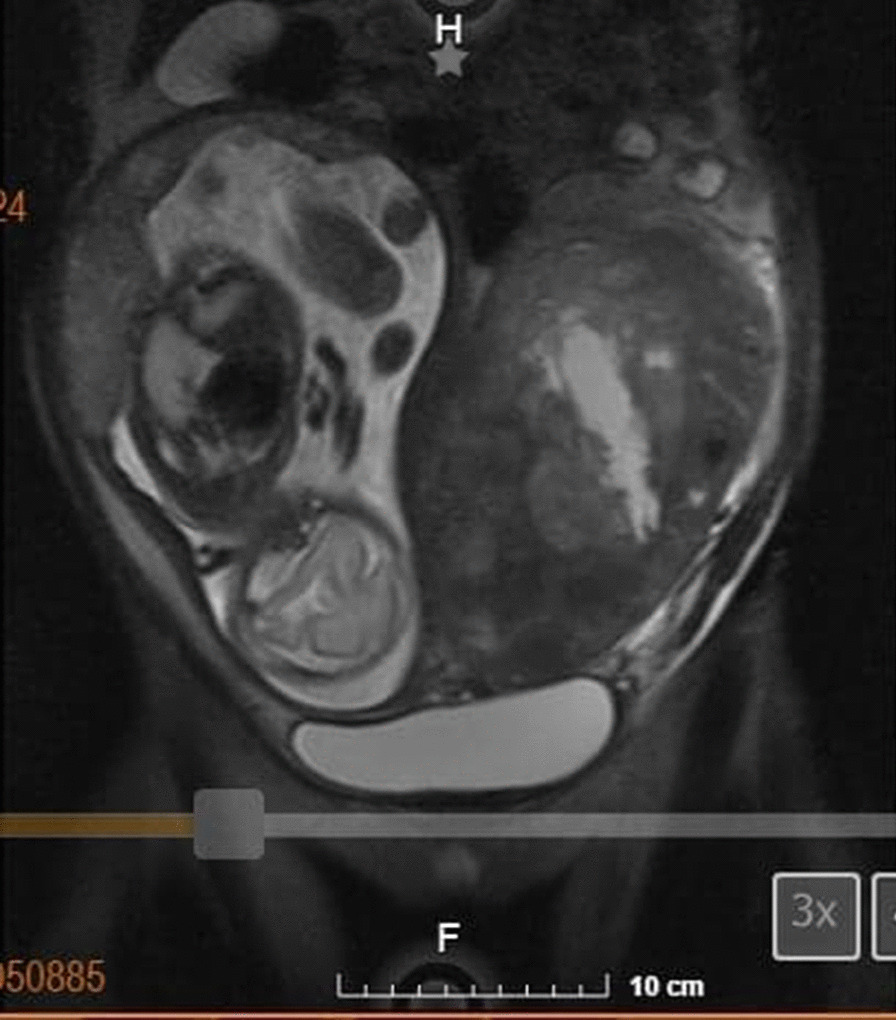
Abdominal-pelvic magnetic resonance imaging, T1 sequence. Pregnancy findings. Image showing one fetus in cephalic presentation, longitudinal lie, fetal dorsum to the left, with normal morphological findings, fundal-lateral placenta of regular margins, and homogeneous parenchyma. Left adnexal mass described in Fig. 2

**Fig. 2:**
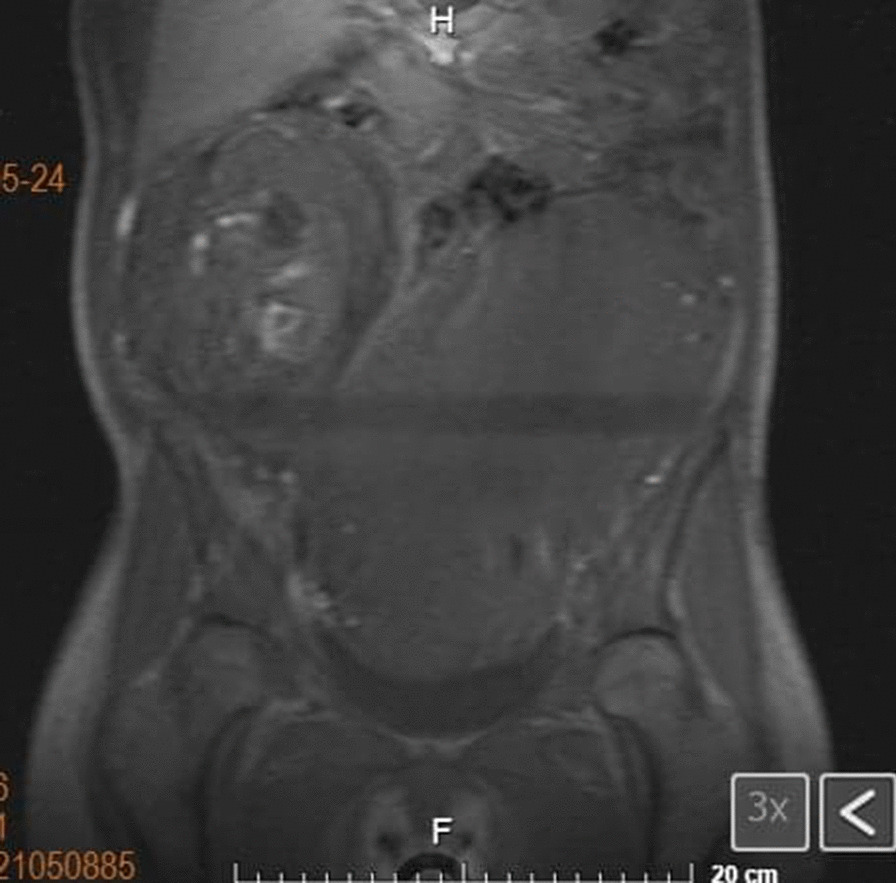
Abdominal-pelvic magnetic resonance imaging, fat-saturated T2-weighted sequence. Findings of the adnexal mass. Coronal view shows a heterogeneous left adnexal lesion, with regular lobulated contours, predominantly isointense to soft tissue, with approximate dimensions of 22.1 × 13.6 × 16.3 cm in its largest diameters

**Fig. 3:**
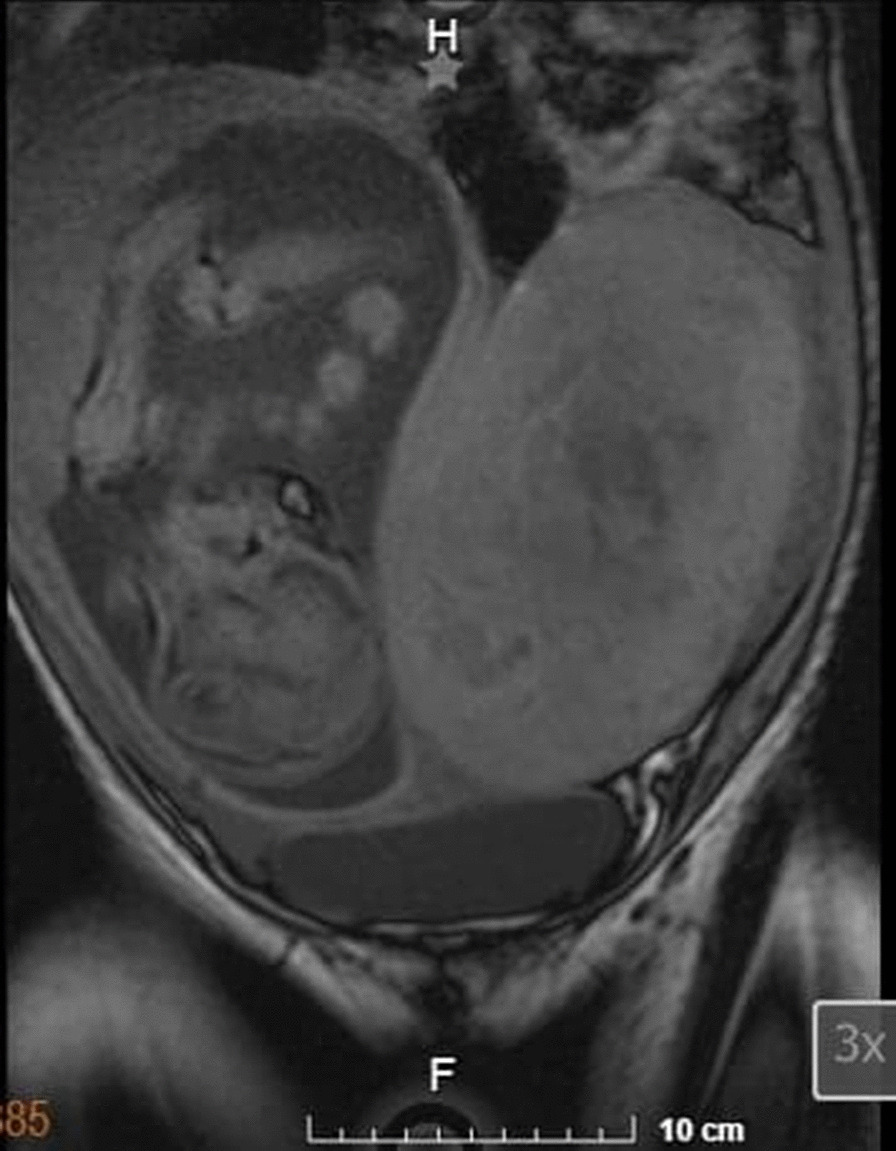
Abdominal-pelvic magnetic resonance imaging, T1- and T2-weighted sequences. Mass effect findings. Image shows left adnexal mass in extensive contact with the uterine corpus that conditions right confinement of the uterus, eccentric displacement of the small intestine, mild compression of the bladder without infiltration, and left ureteral compression

The patient had no relevant past medical history. At 13 weeks of gestation, she was admitted to the hospital due to vaginal bleeding and treated for abruptio placentae, with discharge at recovery. Then at her routine prenatal visit for her 20th (*T*_0_) week of pregnancy, she was found to have a uterine fundus greater than her gestational age, for which she was referred to our hospital to discard polyhydramnios. A pelvic ultrasound was performed, finding a normal intrauterine pregnancy of 25 gestational weeks with cephalic diameter consistent with gestational age, normal cardiac activity, and an adequate amount of amniotic fluid. The ultrasound showed bilateral enlarged ovaries with heterogeneous echogenicity. Due to these findings, pelvic magnetic resonance imaging (MRI) was performed, which showed occupation by an intrauterine pregnancy (Fig. 1), as well as a left tumoral lesion (Fig. 2) with dimensions of 22.1 × 13.6 × 16.3 cm, with regular lobulated contours which caused right confinement of the uterus, as well as eccentric displacement of the small intestine, peripheral vascular structures, and mild compression of the bladder, without infiltration. There was left ureteral compression with mild pyelo-ureteral dilation, as well as mild compression of the inferior vena cava. The lesion was heterogeneous, predominantly isointense to soft tissue in different sequences, with hypointense areas in T1, hyperintense in T2, and irregular borders. It presented extensive contact with the uterine corpus (Fig. 3). The ovaries were not easily visible.

The patient was provided fetal lung maturation therapy with dexamethasone and scheduled for a cesarean section at 26 weeks of gestation (*T*_1_). During surgery, the uterus was opened using a median incision, and the neonate was obtained without complications. The abdominal cavity showed bilateral tumors compatible with the MRI findings. A hysterectomy with right oophorectomy was performed; the patient was discharged 2 weeks later without complications.

The ovarian tumors were sent to pathology, and the results showed poorly differentiated mucinous adenocarcinoma (WHO III) with extensive SRCs.

Two days after discharge, she presented with nausea and vomiting of gastric content accompanied by abdominal pain. She was admitted to the hospital due to the described symptoms and ascites findings; an abdominal paracentesis was then performed. The ascites fluid analysis showed glucose of 20 mg/dL, proteins 1.28 g/dL, and lactate dehydrogenase 1551 U/L, with the presence of gram-positive cocci. Due to the physical and analytical findings, we initiated management with intravenous fluids and antibiotic therapy.

### History of past illness

There was no relevant past medical history, risk factors, or family history of cancer. The patient lived at home with access to all essential services of water and electricity.

### Physical examination

On admission (*T*_0_), she was cachectic, with abdominal distention due to ascites fluid, bulging flanks, and abdominal pain at profound palpation. A 1 cm dehiscence on the vaginal vault was found on the pelvic examination, with fecal material drainage. Her blood pressure remained constant at 100/70 mmHg.

### Laboratory testing

The laboratory assessments showed decreased hemoglobin levels (8.0 g/dL), hematocrit (25.8 %), albumin (2.04 g/dL), proteins (4.60 g/dL), globulin (2.56 g/dL), sodium (132 mEq/L), and chloride (96 mEq/L). Additional laboratory tests showed mildly elevated platelet count (400.0 × 10^3^ u/L), as well as leukocytes (10.0 × 10^3^ u/L), neutrophils (87%), and potassium (5.30 mEq/L).

As a team decision after postsurgical tumor staging, CA-125 levels were not measured due to the limited utility of this marker in patients with poor prognosis. CA-125 levels are usually collected as a follow-up marker to monitor tumor activity after surgery. From this perspective, the patient had such advanced disease that the team deemed it unnecessary to measure CA-125 levels, as it could increase the patient’s burden [5].

### Imaging examination

Panendoscopy displayed an irregular shape of the gastric antrum, fundus, and body due to a neoplastic lesion located in the greater curvature. The histopathological diagnosis of the biopsied specimen was invasive SRC carcinoma. Colonoscopy showed hemorrhoids in the anal canal and sigmoid colon with extrinsic compression suggestive of adhesions.

**Fig. 4:**
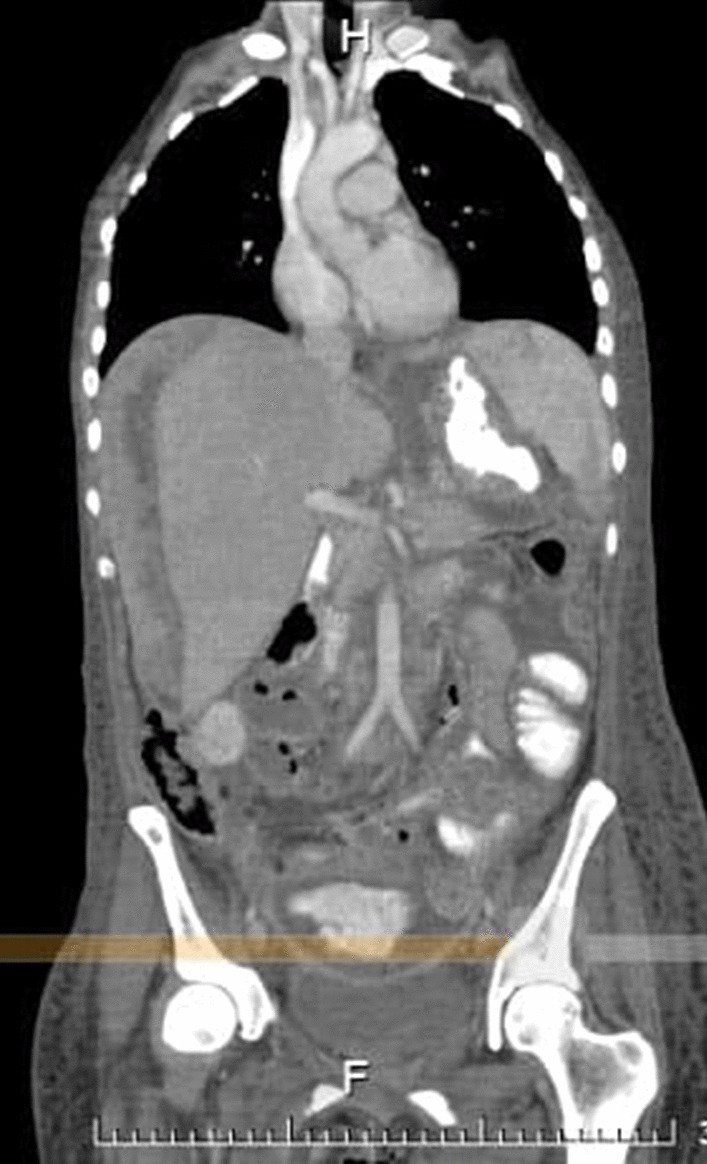
Contrast-enhanced computed tomography of the thorax, abdomen, and pelvis. Postsurgical findings. Image showing bilateral pleural effusion with passive atelectasis of adjacent segments in the left lung, absent uterus due to surgical intervention, and the presence of hydro pneumoperitoneum with air bubbles in the right subphrenic space. An engrossment of the gastric fundus is apparent

Computed tomography (CT) examination of the thorax, abdomen, and pelvis with oral contrast (Fig. 4) showed bilateral pleural effusions, morphological changes in the stomach associated with the diagnosis of gastric cancer, free air, and fluid levels in the peritoneal cavity, with contrast enhancement suggestive of perforation at the anterior wall of the rectum; inflammatory and ischemic changes in the left kidney were also observed.

## Final diagnosis

According to the symptoms, physical examination, imaging, and histopathology findings, this patient was diagnosed with gastric mucinous adenocarcinoma with metastases to the ovary, commonly known as KT. The patient received a poor prognosis due to tumor extension and was offered palliative treatment, which consisted of no further surgeries, providing psychological and emotional support, and pharmacological intervention with opioids for pain management. As she attended a public hospital, there was no financial burden on the patient or the family.

## Treatment

Intervention with exploratory laparotomy yielded the following findings: abdominal sepsis, intestinal perforation, and peritoneal carcinomatosis.

## Outcome and follow-up

After the surgical findings, oncology deferred treatment due to peritoneal involvement, intestinal carcinomatosis, and unresectable gastric cancer. The patient was given palliative therapy and died 2 months later (*T*_2_).

## Summary of clinical course

- *T*_0_: the patient was admitted to the hospital due to a discrepancy between uterus size and gestational age.
- *T*_1_: a cesarean section followed by a hysterectomy with right oophorectomy was performed. The abdominal cavity showed bilateral tumors. Pathology results showed gastric mucinous adenocarcinoma with metastases to the ovary.
- *T*_2_: due to the disease progression, the patient received a poor prognosis and was offered palliative care.

## Discussion

Ovarian cancers occur in approximately 2.8–11 per 100,000 pregnancies. KT represents only 1–2% of these cancers. Approximately 76% of KTs originate from the stomach, 11% from the intestine, 4% from the breast, 3% from the appendix, and the remaining from diverse sites [1, 6, 7].

Sex hormones during pregnancy promote the development and diffusion of gastric cancer by stimulating the underlying precancerous lesions. Placental growth factor levels are high in gastric cancer tissue and are also associated with serosal invasion, lymph node metastasis, cancer stage, and survival rates [8].

In the described case, KT diagnosis presented a challenge. Notably, the patient did not present risk factors for gastric cancer: young non-smoking female, without prior *Helicobacter pylori* infection, and asymptomatic before pregnancy. It has been reported that persistent gastrointestinal symptoms and the physiological and hormonal changes during pregnancy usually mask KT presentation [9].

As recommended in the Guidelines for Diagnostic Imaging During Pregnancy and Lactation from the American College of Obstetricians and Gynecologists (ACOG), once the incidental ultrasound findings were inconclusive, we performed an MRI to further study the suggestive images while avoiding the teratogenic threshold radiation dose (5 to 15 rad).

Optimal treatment for synchronous pregnancy with KT of gastric origin is yet to be established. The available options for treating this neoplasm are cytoreductive surgery (CRS), adjuvant chemotherapy (CTx), neoadjuvant CTx, and hyperthermic intraperitoneal chemotherapy (HIPEC). These treatments may be used alone or in combination [10].

In this patient, CRS was performed, which is the treatment option most associated with an increase in overall survival (OS). In 2019, Lionetti and colleagues conducted a systematic review of the literature and concluded that CRS—particularly CRS in the absence of residuals—presented the clearest results in improving OS in KT patients [10].

Our patient was not treated with CTx. Even though its use is still controversial, some authors recommend CRS + HIPEC as a therapeutic combination, with survival benefits showing more than acceptable morbidity and mortality rates [7, 11].

Despite CRS being performed, oncology deferred further treatment due to peritoneal involvement, intestinal carcinomatosis, and unresectable gastric cancer. We identified ascites, carcinomatosis at the exploratory laparotomy, and the lack of radical surgery for primary cancer as unfavorable prognostic factors that have been associated with poor maternal 2-year survival rates [12, 13].

Kodama and coworkers conducted a study among pregnant women with KT treated with radical surgery (57.1%) or no surgery/palliative surgery (42.9%). The overall maternal survival rate was insufficient in both groups, exhibiting 1, 2, and 5-year rates of 45.6%, 45.6%, and no survival after 5 years [12].

To the best of our knowledge, this is the first case report of a KT diagnosed during pregnancy in Yucatan. More descriptive studies are needed to construct an epidemiological characterization of this cancer in this Mexican region to provide effective therapeutic interventions. The limitation of our study lies in the description of an isolated case of KT, limiting absolute conclusions regarding the standard of care and prognosis of this condition.

## Conclusion

This case report depicts an uncommon example of a young pregnant woman without identifiable risk factors for gastric cancer who manifested a KT. This incidental finding suggests that pregnancy obscured the cancer’s clinical appearance, and the patient’s rapid deterioration corresponded to what is described in the literature. The limited information regarding this neoplasm in Mexico and the torpid evolution of the case highlight the importance of conducting additional studies to generate therapeutic interventions aimed at increasing overall survival.

## Data Availability

The datasets used and/or analyzed during the current study are available from the corresponding author on reasonable request.

## Abbreviations

KT: Krukenberg tumor
WHO: World Health Organization
SRC: Signet ring cells
MRI: Magnetic resonance imaging
CT: Computed tomography
ACOG: American College of Obstetricians and Gynecologists
CRS: Cytoreductive surgery
CTx: Adjuvant chemotherapy
HIPEC: Hyperthermic intraperitoneal chemotherapy
OS: Overall survival
*T*_0_: Time of initial presentation
*T*_1_: Time of next follow-up
*T*_2_: Time of last intervention
